# Effect of Copper on Expression of Functional Genes and Proteins Associated with *Bradyrhizobium diazoefficiens* Denitrification

**DOI:** 10.3390/ijms23063386

**Published:** 2022-03-21

**Authors:** Pedro J. Pacheco, Juan J. Cabrera, Andrea Jiménez-Leiva, Eulogio J. Bedmar, Socorro Mesa, Germán Tortosa, María J. Delgado

**Affiliations:** Department of Soil Microbiology and Symbiotic Systems, Estación Experimental del Zaidín, Consejo Superior de Investigaciones Científicas, 18008 Granada, Spain; pedro.pacheco@eez.csic.es (P.J.P.); juan.cabrera@eez.csic.es (J.J.C.); andrea.jimenez@eez.csic.es (A.J.-L.); eulogio.bedmar@eez.csic.es (E.J.B.); socorro.mesa@eez.csic.es (S.M.); german.tortosa@eez.csic.es (G.T.)

**Keywords:** Cu-containing nitrite reductase, enzymatic activity, gene expression, nitric oxide reductase, nitrous oxide reductase, periplasmic nitrate reductase

## Abstract

Nitrous oxide (N_2_O) is a powerful greenhouse gas that contributes to climate change. Denitrification is one of the largest sources of N_2_O in soils. The soybean endosymbiont *Bradyrhizobium diazoefficiens* is a model for rhizobial denitrification studies since, in addition to fixing N_2_, it has the ability to grow anaerobically under free-living conditions by reducing nitrate from the medium through the complete denitrification pathway. This bacterium contains a periplasmic nitrate reductase (Nap), a copper (Cu)-containing nitrite reductase (NirK), a *c*-type nitric oxide reductase (cNor), and a Cu-dependent nitrous oxide reductase (Nos) encoded by the *napEDABC, nirK, norCBQD* and *nosRZDFYLX* genes, respectively. In this work, an integrated study of the role of Cu in *B. diazoefficiens* denitrification has been performed. A notable reduction in *nirK*, *nor,* and *nos* gene expression observed under Cu limitation was correlated with a significant decrease in NirK, NorC and NosZ protein levels and activities. Meanwhile, *nap* expression was not affected by Cu, but a remarkable depletion in Nap activity was found, presumably due to an inhibitory effect of nitrite accumulated under Cu-limiting conditions. Interestingly, a post-transcriptional regulation by increasing Nap and NirK activities, as well as NorC and NosZ protein levels, was observed in response to high Cu. Our results demonstrate, for the first time, the role of Cu in transcriptional and post-transcriptional control of *B. diazoefficiens* denitrification. Thus, this study will contribute by proposing useful strategies for reducing N_2_O emissions from agricultural soils.

## 1. Introduction

With a 300-fold greater global warming potential than carbon dioxide (CO_2_), nitrous oxide (N_2_O) is one of the main biogenic greenhouse gases (GHG), and has also been described as the biggest single cause of ozone depletion [1]. N_2_O emissions from human activities, fundamentally Agriculture, Forestry and Other Land Use (AFOLU), have notably increased since the Green Revolution in the early 60 s. During the period 2007–2016, these activities represented 81% of the anthropogenic emissions of N_2_O, according to the last special report by the Intergovernmental Panel on Climate Change [2]. In particular, agriculture has become the major source of N_2_O emissions, accounting for approximately 78% of the anthropogenic N_2_O sources [2] because of a global agricultural intensification and a great increase in the non-synchronized use of synthetic nitrogen fertilisers [3,4,5]. Several biological pathways occurring in agricultural soils are involved in N_2_O emissions. Among all of them, nitrification and denitrification are the main microbial N_2_O sources directly affected by soil nitrogen fertilisation, but only denitrification is known to be the largest source of N_2_O [6]. 

Apart from other organisms, such as archaea and fungi, some facultative bacteria possess the ability to adapt their metabolism to an oxygen-depleted environment in the presence of nitrate as a respiratory substrate through the activation of denitrification. This pathway consists of the dissimilatory reduction of nitrate (NO_3_^−^) or nitrite (NO_2_^−^) to dinitrogen (N_2_) via the gaseous intermediates nitric oxide (NO) and nitrous oxide (N_2_O). In this process, specific metalloenzymes are sequentially involved: periplasmic (Nap) or membrane-bound (Nar) nitrate reductases, copper (Cu)-containing (NirK) or cytochrome *cd*_1_-containing (NirS) nitrite reductases, nitric oxide reductases (cNor, qNor or Cu_A_Nor), and nitrous oxide reductase (Nos). The majority of denitrifiers are found in the phylum *Proteobacteria,* within the domain *Bacteria* [7]. The α-proteobacterium *Paracoccus denitrificans* and the γ-proteobacteria *Pseudomonas stutzeri* and *Pseudomonas aeruginosa* are the first model organisms where denitrification were widely studied. Reviews covering the physiology, biochemistry and molecular genetics of denitrification have been published elsewhere [8,9,10,11,12,13,14].Over recent years, several reports about denitrification in plant endosymbiotic bacteria emerged [15,16,17]. Thanks to their capacity to establish an N_2_-fixing symbiotic relationship with plants, these bacteria can contribute to natural N soil enrichment, while reducing the need for chemical fertilisation. Therefore, symbiotic N_2_ fixation is considered a process with economic, ecological and agricultural importance. In this process, a mutualist association between soil bacteria, commonly known as rhizobia, and plants of the Fabaceae family is established. Rhizobia may induce the formation of nodules in the legume roots and on the stems of some aquatic legumes; nodules are specialized structures where N_2_ fixation takes place [18].

*Bradyrhizobium diazoefficiens*, which establishes nitrogen-fixation symbiosis with soybean (*Glycine max*), is considered a model organism in the study of denitrification in rhizobia because it is the only known rhizobia species able to grow under oxygen-limiting conditions with NO_3_^−^ as sole electron acceptor and, also, to perform the complete denitrification pathway under both free-living and symbiotic conditions [15]. Denitrification in *B. diazoefficiens* is carried out by a periplasmic nitrate reductase (Nap), encoded by the *napEDABC* operon [19], a Cu-containing nitrite reductase (NirK), encoded by the *nirK* gene [20], a cytochrome *c*-type nitric oxide reductase (cNor), encoded by the *norCBQD* operon [21], and a Cu-dependent nitrous oxide reductase (Nos), encoded by the *nosRZDFYLX* genes [22]. Nap is a functional heterodimer comprising the catalytic subunit NapA of about 90 kDa that contains a *bis* molybdopterin guanine dinucleotide (Mo[MGD]_2_) cofactor and a [4Fe-4S] centre, and NapB (15 kDa) that contains 2 heme *c* groups and receives electrons from the membrane-bound NapC (25 kDa) which binds 4 heme *c* groups. NirK is a homotrimer with a predicted molecular mass of about 35 kDa per monomer that contains type 1 and type 2 Cu centres. The catalytic subunit of cNor, NorB, contains heme *b* and a binuclear active centre (heme *b*_3_ and Fe_B_). NorC is a membrane-anchored protein (16 kDa) that contains heme *c*. Finally, the catalytic subunit of Nos, NosZ (120–160 kDa), is a homodimer Cu-containing enzyme with two distinct Cu centres (Cu_A_ and Cu_Z_). 

Similarly to many other denitrifiers, expression of denitrification genes in *B. diazoefficiens* requires both oxygen limitation and the presence of nitrate or a derived nitrogen oxide (NOx), this control being mediated by the FixLJ-FixK_2_-NnrR regulatory cascade [23,24,25]. In fact, the expression of *napEDABC, nirK* and *nosRZDFYLX* genes requires microoxic conditions and directly depends on the transcriptional regulator FixK_2_ [25,26], while expression of *norCBQD* genes relies on NO, being NnrR the transcriptional regulator which directly interacts with the *norCBQD* promoter [25,27]. In this context, the molecular discriminatory determinants for selective FixK_2_ recognition and target activation were recently unveiled [28].

Besides being a source of N_2_O, the ecological and environmental importance of denitrification lies in the fact that Nos is the only known enzyme able to remove N_2_O from ecosystems [4], the expression and activity of this enzyme becoming a natural target to effectively reduce N_2_O emissions from agricultural soils. Increasing knowledge of the regulation and biochemistry of N_2_O metabolism in rhizobia will raise opportunities for the design of effective mitigation strategies to reduce N_2_O emissions from legume crops [6,14,29]. 

Nowadays, new environmental factors are emerging as candidates for controlling denitrification, such as pH [30,31] or Cu [32]. In the case of Cu, it is an essential cofactor in critical enzymes, such as multicopper oxidases, as well as the Nos and NirK denitrification enzymes. The role of this metal in denitrification has been studied in a wide range of non-symbiotic microorganisms, such as *Pseudomonas perfectomarinus* [33], *P. stutzeri* [32], *P. denitrificans* [34,35] and *Achromobacter xylosoxidans* [34]. Regarding rhizobia, Serventi et al. (2012) [36] investigated the role of Cu in cytochrome oxidase biogenesis in *B. diazoefficiens.* Nevertheless, studies covering Cu influence on the denitrification pathway in rhizobia are scarce. This study provides an integral view of the involvement of Cu in *B. diazoefficiens* denitrification, analysing the effect of different Cu regimes on gene expression, as well as on the protein levels and activity of the denitrification enzymes in free-living cultures. 

## 2. Results

### 2.1. Copper Effect on B. diazoefficiens 110spc4 Growth under Different Oxygen Conditions

**Figure 1:**
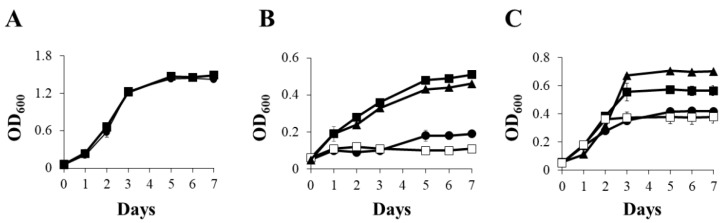
Growth of *B. diazoefficiens* 110*spc*4 in Cu limitation (Cu-L) (●), Cu standard (Cu-S) (■) and high Cu (Cu-H) (▲) BVMN media under oxic (**A**), anoxic (**B**) and microoxic (**C**) conditions. In (**B**,**C**), growth in the Cu-S BVM medium was also included (□). Error bars represent standard error between triplicates, and where not visible, these were smaller than the symbols.

*B. diazoefficiens* 110*spc*4 was grown under oxic, anoxic and microoxic (2% initial O_2_ concentration) conditions in Buffered Vincent’s medium [36] supplemented with nitrate (BVMN) and different Cu concentrations: Cu limitation, i.e., chelated (Cu-L), Cu standard (Cu-S, 0.02 µM) or high Cu (Cu-H, 13 µM) (Figure 1). Under oxic conditions, cultures reached an optical density at 600 nm (OD_600_) of around 1.5 after 7 days of incubation, regardless of the Cu treatment (Figure 1A). These results suggest that Cu was not a limiting factor for *B. diazoefficiens* growth by oxygen respiration (Figure 1A). 

When *B. diazoefficiens* 110*spc*4 cells were cultured in BVMN medium under anoxic conditions (Figure 1B), Cu-L cultures reached a turbidity (OD_600_) of about 0.2 after 7 days of incubation, while Cu-S and Cu-H cultures reached an OD_600_ of about 0.5, indicating that growth was severely affected in the Cu-L medium compared with Cu-S or Cu-H media (Figure 1B). This result indicates that Cu was essential for nitrate-dependent anaerobic growth of *B. diazoefficiens*. In fact, the growth profile displayed in BVMN Cu-L cultures with NO_3_^−^ was similar to that observed in Cu-S cultures incubated without NO_3_^−^ (BVM medium) (Figure 1B), indicating that Cu and NO_3_^−^ were both indispensable for NO_3_^−^ respiration under anoxic conditions. Finally, *B. diazoefficiens* 110*spc*4 cells were incubated under microoxic conditions in Cu-L, Cu-S and Cu-H BVMN media. As shown in Figure 1C, microaerobic growth under Cu-L conditions decreased compared with that reached under Cu-S conditions (about 0.4 and 0.6 OD_600_, respectively, after 7 days of incubation). In contrast, cells grown in the Cu-H medium showed a significant increase in growth rates compared with those cultured in the Cu-S medium (about 0.7 and 0.6 OD_600_, respectively, after 7 days of incubation). Interestingly, when cells were grown microaerobically in the Cu-S medium, but in the absence of NO_3_^−^, they displayed similar growth rates to those cultured in Cu-L conditions with NO_3_^−^ as the respiratory substrate (Figure 1C). These results suggest that NO_3_^−^ and Cu were necessary for *B. diazoefficiens* to grow from NO_3_^−^ respiration under microoxic conditions, as it was observed under anoxic conditions (Figure 1B). 

### 2.2. Disparate Response of Denitrification Gene Expression to Copper 

A preliminary experiment was performed in order to select the incubation period in which denitrification gene induction in response to oxygen depletion in a BVMN medium reached maximal levels. To achieve this goal, β-galactosidase activity from a *napE-lacZ*, *nirK-lacZ*, *norC-lacZ* and *nosR-lacZ* transcriptional fusions was analysed in *B. diazoefficiens* parental cells grown in Cu-S BVMN medium under oxic and microoxic conditions for 1, 2 and 3 days of incubation. As shown in Figure S1, all fusions showed a gradual increase in β-galactosidase activity from 1 to 3 days of growth. In general, microaerobic expression of all denitrification genes was notably higher than that observed under aerobiosis. These results confirmed previous studies [15,25], where oxygen-limiting conditions together with NO_3_^−^ strongly induced denitrification gene expression in *B. diazoefficiens*. In contrast to the remarkably low levels of aerobic expression of *nap*, *nirK* and *nor* genes, the *nosR-lacZ* fusion showed significantly higher levels of β-galactosidase activity after 1, 2 and 3-day incubation periods under oxic conditions. Similar levels of *nosR*-*lacZ* expression were observed under aerobic and microaerobic respiration after 1 day of incubation. However, a significant induction of β-galactosidase activity was observed from the *nosR*-*lacZ* fusion in cells incubated for 2 or 3 days under microoxic conditions compared with that from aerobically grown cells (Figure S1). 

**Figure 2:**
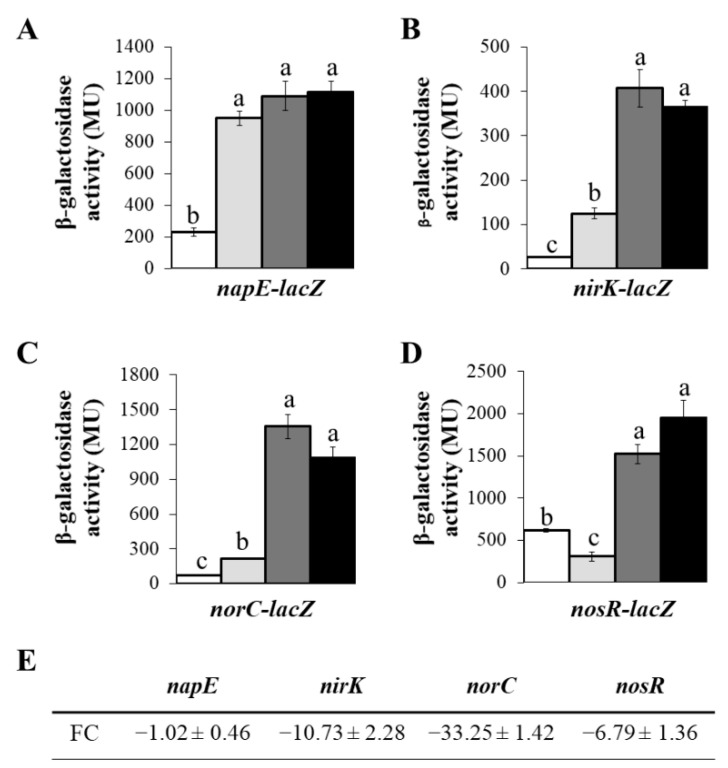
Transcriptional expression of denitrification genes monitored as β-galactosidase activity from *napE-lacZ* (**A**), *nirK-lacZ* (**B**), *norC-lacZ* (**C**) and *nosR-lacZ* (**D**) fusions chromosomally integrated in *B. diazoefficiens* 110*spc*4 grown aerobically in Cu-S (white bars) and microaerobically in Cu-L (light grey bars), Cu-S (dark grey bars) and Cu-H (black bars) BVMN media for 3 days. A *post-hoc* Tukey HSD test at *p* ≤ 0.05 was applied in (**A**–**D**); same lower-case letters in each figure indicate that values are not statistically different. (**E**) Expression changes of *napE*, *nirK*, *norC* and *nosR* genes in *B. diazoefficiens* 110*spc*4 grown microaerobically in Cu-L compared with Cu-S measured by qRT-PCR. Data expressed as Miller Units (MU) and Fold Change (FC) are means with standard deviation from at least three independent cultures assayed in triplicate.

Taking these results into consideration, we decided to perform a study into the Cu effect on denitrification gene expression, by incubating cells for 3 days under different Cu concentrations. For this purpose, we analysed β-galactosidase activity from the *napE-lacZ*, *nirK-lacZ*, *norC-lacZ* and *nosR-lacZ* transcriptional fusions in *B. diazoefficiens* 110*spc*4 cells grown for 3 days in Cu-L, Cu-S and Cu-H BVMN media, under microoxic conditions (Figure 2). Cultures grown aerobically were included as a control in the experiments. As shown in Figure 2A, *napE-lacZ* microaerobic expression was not significantly affected by Cu concentration in the medium, demonstrating similar β-galactosidase activity values under Cu-L, Cu-S or Cu-H conditions. These results indicate that Cu is not involved in the transcriptional control of *napEDABC* genes. In contrast, Cu limitation drastically lowered β-galactosidase activity from the *nirK-lacZ*, *norC-lacZ* and *nosR*-*lacZ* fusions (about 3-, 6- and 4-fold, respectively) compared with the values obtained in the Cu-S medium (Figure 2B–D). These results suggest that Cu availability is essential for *nirK*, *norCBQD* and *nosRZDFYLX* maximal expression. Since the catalytic subunit of cNor, NorB, does not contain Cu as cofactor, the drastic reduction in *norCBQD* expression under Cu-L conditions, instead of being directly controlled by Cu, might be due to the lack of nitric oxide (NO), the product of NirK, which is required to induce the expression of *norCBQD* genes [25,27]. In order to test this hypothesis, we performed a β-galactosidase activity assay with *B. diazoefficiens* cells containing the *norC-lacZ* fusion incubated under Cu-L and Cu-S BVM media (i.e., without NO_3_^−^) in the presence of NO that was added to the cells 5 h before the assay. As shown in Figure S2, the addition of NO to the Cu-L BVM medium resulted in a very weak induction of about 3-fold of *norC-lacZ* expression, in contrast to the notable increase of about 9.5-fold in the NO-amended Cu-S BVM medium (Figure S2). Thus, the decreased expression of *norCBQD* genes under Cu limitation was not due to the lack of NO provoked by the reduction in *nirK* expression under Cu-L conditions, but rather to a direct unknown effect of Cu limitation on *norC-lacZ* expression. Regarding the effect of high Cu concentrations on denitrification gene expression, the growth of the cells in the Cu-H BVMN medium did not change the expression of any of the transcriptional fusions significantly, compared to that observed in cells grown in the Cu-S BVMN medium (Figure 2A–D).

The negative effect of Cu limitation on *nirK*, *nor* and *nos* transcriptional expression was also confirmed by qRT-PCR analyses. When cells were cultured microaerobically in the Cu-L BVMN medium, expression of *nirK*, *norC* and *nosR* genes was reduced to 10.73, 33.25 and 6.79, respectively, compared with that observed in cells cultured in the Cu-S medium (Figure 2E). In contrast, Cu limitation did not affect *napE* expression compared with Cu-S conditions, similar to the results obtained when we analysed the *napE*-*lacZ* transcriptional fusion (Figure 2A,E). Taken together, these results confirm the negative effect of Cu limitation on *nirK*, *nor* and *nos* but not on *nap* gene expression. 

### 2.3. Influence of Copper on Expression and Activity of Denitrification Enzymes 

**Figure 3:**
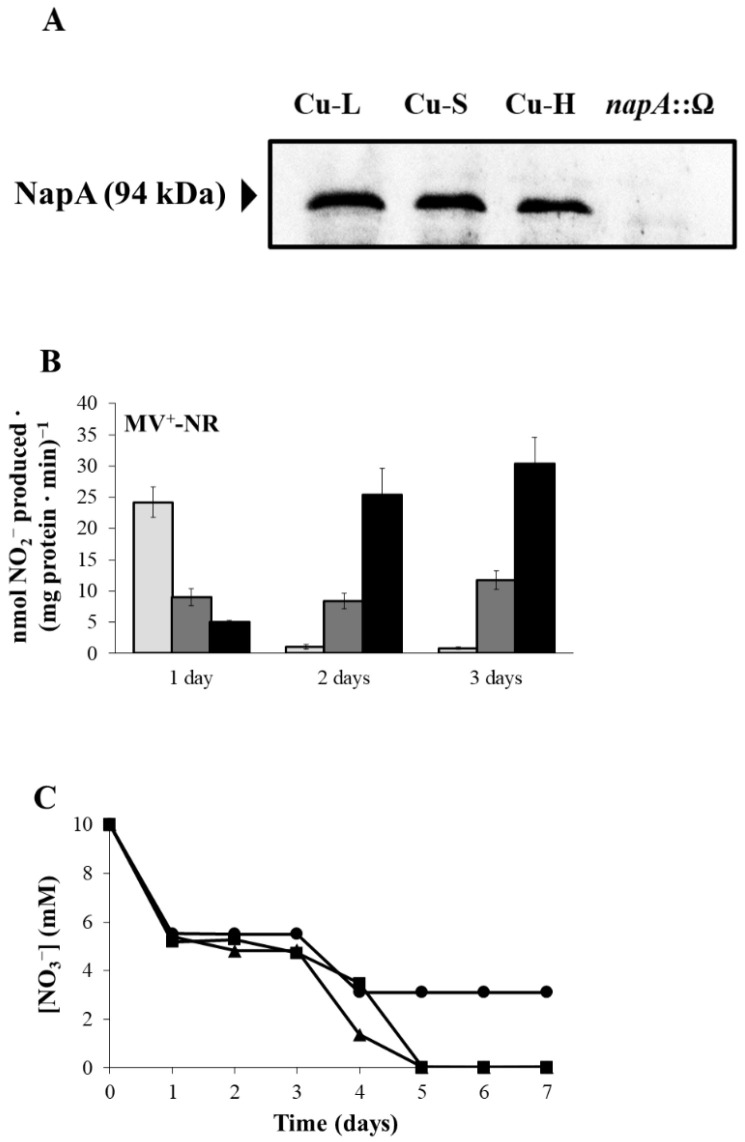
Nitrate reductase protein levels and activity. (**A**) Western-blotted sodium dodecyl sulphate gel electrophoresis (SDS-PAGE) gels of soluble fraction (20 µg) proteins probed with anti-NapA antibodies from *P. pantotrophus*. Soluble fraction from a *napA*::Ω mutant strain was used as negative control in the experiments. Apparent mass of NapA (94 kDa) is shown in the left margin. Soluble fraction was isolated from 3-day incubation cultures. (**B**) Methyl viologen-dependent nitrate reductase (MV^+^-NR) activity in Cu-L (light grey bars), Cu-S (dark grey bars) and Cu-H (black bars) conditions. Data are means with standard error bars from at least two independent cultures assayed in triplicate. (**C**) Extracellular nitrate concentration in the Cu-L (●), Cu-S (■) and Cu-H (▲) growth media. Error bars represent standard error between triplicates, and where not visible, these were smaller than the symbols. Cells were grown under microoxic conditions in BVMN medium with different Cu concentrations.

In order to investigate the effects of Cu on Nap expression, immunoblot analyses using antibodies raised against purified *Paracoccus pantotrophus* NapA [37] were performed in the soluble fraction of *B. diazoefficiens* grown under microoxic conditions in Cu-L, Cu-S and Cu-H BVMN media. A band of about 94 kDa, that was undetectable in a *napA* mutant, was present in the wild-type (WT) cells (Figure 3A). Expression of NapA was not affected by Cu since similar protein levels were observed in WT cells grown under the different Cu conditions assayed (Figure 3A). Full scans of the entire gel from a representative experiment are shown in Figure S3A,B. Next, methyl viologen-dependent nitrate reductase (MV^+^-NR) activity was analysed in *B. diazoefficiens* parental cells incubated for 1, 2 and 3 days under microoxic conditions in Cu-L, Cu-S or Cu-H BVMN media. As shown in Figure 3B, MV^+^-NR activity in Cu-L conditions was higher than that detected in Cu-S or Cu-H media after 1 day of incubation. However, MV^+^-NR activity was much lower than that detected in Cu-S or Cu-H media after 2 and 3 days of incubation. Low levels of MV^+^-NR activity observed after 2 and 3 days of incubation in Cu-L conditions were in concordance with nitrate consumption rates, where only 7 mM NO_3_^−^ was taken from the medium, while all NO_3_^−^ present (10 mM) was consumed by the cells grown under Cu-S and Cu-H conditions after 5 days of incubation (Figure 3C). It is also important to note that the highest values of MV^+^-NR activity were obtained in Cu-H medium either after 2 or 3 days of incubation (Figure 3B). In fact, while 8.7 mM NO_3_^−^ was consumed by cells grown under Cu-H conditions, only 6.6 mM NO_3_^−^ was taken by Cu-S grown cells after 4 days of incubation (Figure 3C). 

**Figure 4:**
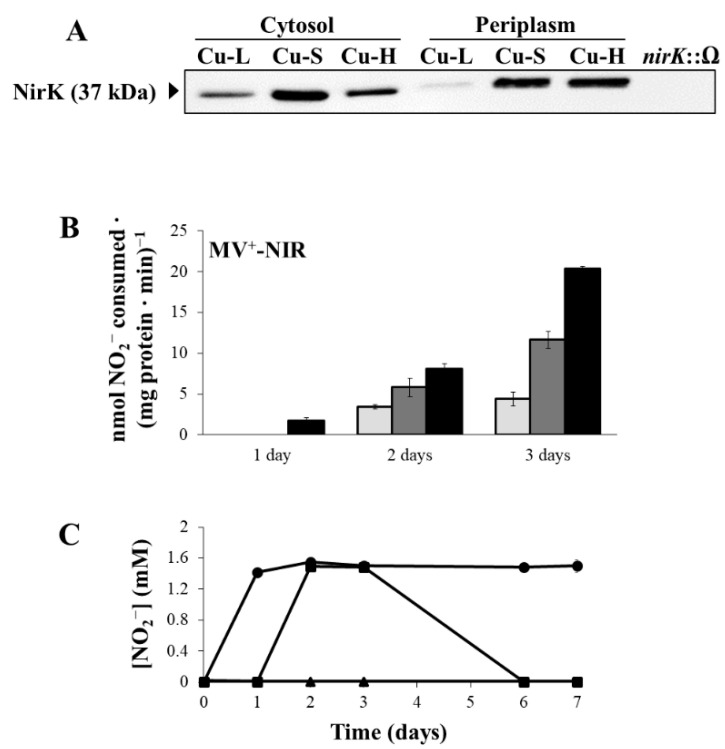
Nitrite reductase protein levels and activity. (**A**) Western-blotted SDS-PAGE gels of periplasmic (21 µg) and cytosolic (12 µg) proteins probed with anti-NirK antibodies from *B. diazoefficiens* 110*spc*4. Soluble fraction from a *nirK*::Ω mutant strain was used as negative control in the experiments. Apparent mass of NirK (37 kDa) is shown in the left margin. Periplasmic and cytosolic fractions were isolated from 3-day incubation cultures. (**B**) Methyl viologen-dependent nitrite reductase (MV^+^-NIR) activity under Cu-L (light grey bars), Cu-S (dark grey bars) and Cu-H (black bars) conditions. Data are means with standard error bars from at least two independent cultures assayed in triplicate. (**C**) Extracellular nitrite (NO_2_^−^) concentration in the Cu-L (●), Cu-S (■) and Cu-H (▲) growth media. Error bars represent standard error between triplicates, and where not visible, these were smaller than the symbols. Cells were grown in BVMN medium with different Cu concentrations under microoxic conditions.

Figure 4A displays NirK western blot analyses in cytosolic and periplasmic fractions. The band corresponding to NirK (37 kDa), that was absent in a *nirK* mutant, was fainter in the cytosol (2.3-fold) and very weakly detectable in the periplasm (4.5-fold lower expression) from cells grown under Cu-L conditions compared with that detected in cells grown in the Cu-S medium (Figure 4A). In contrast, while the levels of NirK in the cytosol of Cu-H cells were 1.8-fold lower than those detected in Cu-S cells, a similar pattern of expression was observed in the periplasm of the Cu-H- and Cu-S-grown cells (Figure 4A). Full scans of the entire gel from a representative experiment are shown in Figure S4A,D. With respect to NirK activity, methyl viologen-dependent nitrite reductase (MV^+^-NIR) activity levels were significantly lower in the Cu-L than in the Cu-S medium, mainly after 3 days of incubation (Figure 4B). This observation was consistent with the accumulation of NO_2_^−^ in the Cu-L medium (1.5 mM NO_2_^−^) that was not further consumed through the entire 7-day incubation period (Figure 4C). In contrast, Cu-S-grown cells consumed the NO_2_^−^ accumulated (1.5 mM NO_2_^−^) from the third day of incubation, decreasing its concentration in the growth medium to zero after 6 days (Figure 4C). As observed in Figure 4C, NO_2_^−^ was accumulated in the Cu-L medium from the first day, indicating a possible inhibitory effect on Nap activity, observed after 2 and 3 days of incubation (see Figure 3B). To confirm this hypothesis, we analysed NO_2_^−^ accumulation and MV^+^-NR activity in cells of a *nirK* mutant that, as has been reported previously, also accumulates NO_2_^−^ in the growth medium [20,38]. For this purpose, WT and *nirK* mutant cells were cultured for 3 days in Cu-L or Cu-S media. As expected, NO_2_^−^ accumulated in the WT Cu-L medium, as well as in the *nirK* Cu-L and Cu-S media (Figure S5A). Moreover, very low MV^+^-NR activity values were observed in WT under Cu-L and in the *nirK* mutant both under Cu-L and Cu-S conditions, compared with those found in WT grown in the Cu-S medium (Figure S5B). 

As shown in Figure 4B, MV^+^-NIR activity was notably higher in cells grown for 3 days under Cu-H conditions compared with that observed under Cu-S conditions (Figure 4B). In fact, NO_2_^−^ was not detected in the Cu-H medium throughout the entire incubation period (Figure 4C). This observation might be a consequence of the high NIR activity observed under Cu-H conditions (Figure 4B). 

**Figure 5:**
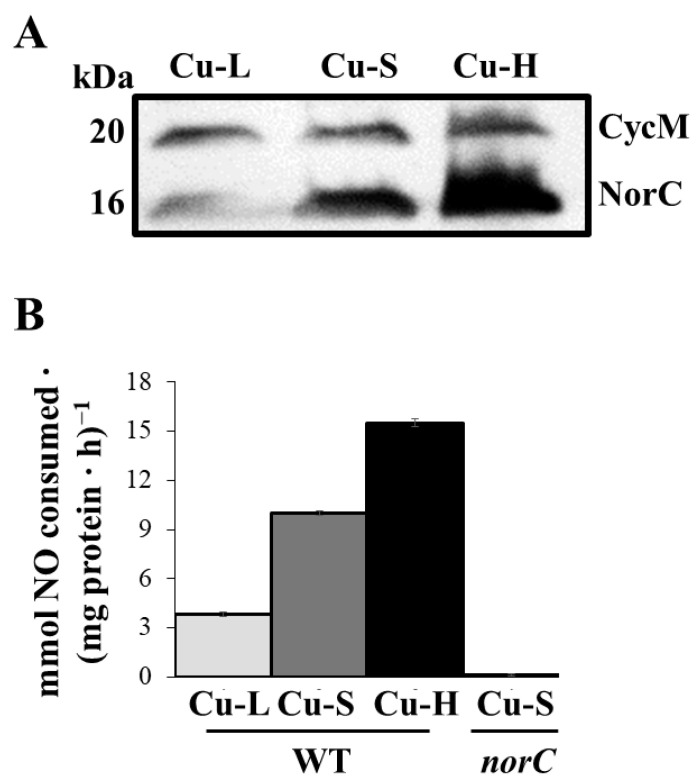
Nitric oxide reductase expression and activity. (**A**) Heme-stained proteins (30 µg) of membranes prepared from *B. diazoefficiens* 110*spc*4. CycM and NorC cytochromes identified previously are specified in the right margin. Apparent masses of the proteins (kDa) are shown in the left margin. (**B**) Nitric oxide reductase activity of *B. diazoefficiens* 110*spc*4 (WT). The *norC::aphII*-PSP (*norC*) mutant strain cultured in Cu-S medium was used as negative control in the experiments. Data represent means with standard error bars from at least two independent cultures assayed in triplicate. Cells were grown microaerobically in Cu-L, Cu-S or Cu-H BVMN medium for 3 days.

Next, we analysed the expression of NorC by heme-staining of the membrane proteins (Figure 5A). The NorC subunit of the nitric oxide reductase (NOR) enzyme, identified previously by Mesa et al. (2002) [21], is about 16 kDa. As observed in Figure 5A, about 3.3-fold lower levels of NorC were detected in cells grown under Cu-L conditions compared with those observed in those grown in Cu-S. Moreover, an approximate 2.3-fold increase in NorC expression was detected in Cu-H compared with Cu-S conditions (Figure 5A). Full scans of the entire gel from a representative experiment are shown in Figure S6A,B. Similarly, NOR activity was notably lower in cells incubated in the Cu-L medium than in those grown under Cu-S conditions (Figure 5B). A significant induction of NOR activity was also observed under Cu-H conditions compared with that from Cu-S grown cells (Figure 5B). A *norC* mutant, which lacked NOR activity, cultured in the Cu-S medium was used as a negative control in the experiments (Figure 5B). 

**Figure 6:**
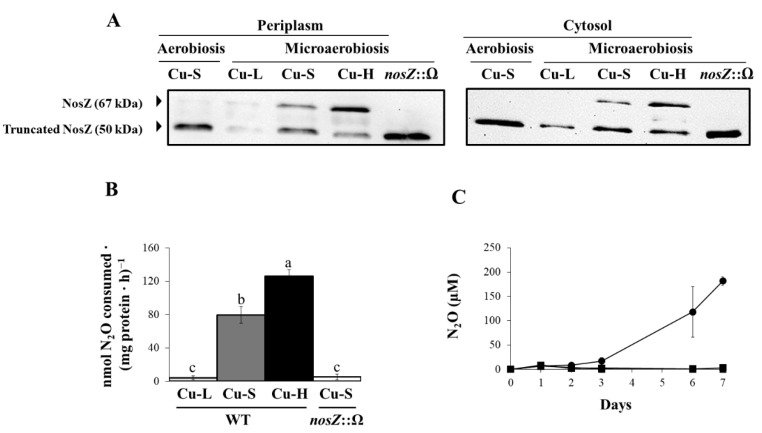
Nitrous oxide reductase expression and activity. (**A**) Western-blotted SDS-PAGE gels of periplasmic (left, 21 µg) and cytosolic (right, 14 µg) proteins probed with anti-NosZ antibodies from *P. denitrificans*. Apparent masses of NosZ (67 kDa) and truncated NosZ (50 kDa) are shown in the left margin. Periplasmic and cytosolic fractions were isolated from 3-day incubation cultures. (**B**) N_2_O consumption capacity of cells grown for 3 days under Cu-L (light grey bar), Cu-S (dark grey bar) and Cu-H (black bar) conditions. Soluble proteins (**A**) and cells (**B**) from a *nosZ*::Ω mutant cultured microaerobically in Cu-S medium were used as negative controls in the experiments. Data represent means with standard error bars from at least two independent cultures assayed in triplicate. In B, a *post-hoc* Tukey HSD test at *p* ≤ 0.05 was applied; same lower-case letters indicate that values are not statistically different. (**C**) Nitrous oxide (N_2_O) accumulation in the headspace of Cu-L (●), Cu-S (■) and Cu-H (▲) growth medium. Error bars represent standard error between triplicates, and where not visible, these were smaller than the symbols. Cells were grown aerobically or microaerobically in BVMN medium with different Cu concentrations.

Finally, we analysed NosZ expression in periplasmic and cytosolic fractions by western blot using an antibody against purified *P. denitrificans* NosZ [34]. As shown in Figure 6A, two bands were observed in periplasm (left) and cytosol (right) of WT cells grown microaerobically in the Cu-S medium. The 67 kDa band corresponds to NosZ and the 50 kDa band corresponds to the C-terminal truncated NosZ protein, as previously reported for NosZ from *B. diazoefficiens* [27] and from *P. denitrificans* [34]. As expected, the 67 kDa band was absent in the *nosZ* mutant which confirmed that such a band corresponds to NosZ. However, a band of about 50 kDa was present in the cytosol and periplasm of the *nosZ* insertion mutant where the C-terminal domain was deleted, producing a shorter polypeptide. NosZ was very faintly expressed under oxic Cu-S conditions, as well as under microoxic Cu-L conditions, in both cellular fractions (Figure 6A). However, NosZ was detected in cells grown under Cu-S and Cu-H conditions, and its concentration increased in the periplasmic (about 2.2-fold) and cytosolic (about 2.3-fold) fractions from cells incubated in the Cu-H medium compared with those grown in the Cu-S medium. Interestingly, the 67 kDa band observed in the periplasm of Cu-H grown cells was 1.9-fold more intense than that of 50 kDa (Figure 6A). In contrast, in the periplasm of Cu-S grown cells, the level of the mature NosZ of 67 kDa was 1.6-fold lower than that of the truncated protein (50 kDa). These results suggest a clear effect of Cu on the maturation of the protein in the periplasm. Full scans of the entire gels from a representative experiment are shown in Figure S4B,C,E,F. Next, nitrous oxide reductase (N_2_OR) activity was measured in cells grown under microoxic Cu-L, Cu-S and Cu-H conditions. The *nosZ* mutant cultured in Cu-S medium was also included as negative control in the experiments. As shown in Figure 6B, N_2_OR activity in the Cu-L medium was diminished to levels similar to those of the *nosZ* mutant. A strong induction of N_2_OR activity was observed in cells grown under Cu-S conditions compared to that from cells under Cu-L conditions. When cells were incubated in the Cu-H medium, an increase in N_2_OR activity of 1.6-fold was observed compared with that from Cu-S grown cells (Figure 6B). Figure 6C shows N_2_O accumulation in the headspace of the medium through 7 days of incubation. Under Cu-L conditions, N_2_O was accumulated from the third day of incubation, increasing its concentration from 16.61 ± 7.36 µM (day 3) to 118.12 ± 51.65 µM (day 7). However, N_2_O was not detected in the headspace of Cu-S or Cu-H cultures throughout the entire incubation period (Figure 6C). 

### 2.4. Investigating the Possible Role of NosR in Nos Gene Expression and Nos Activity

**Figure 7:**
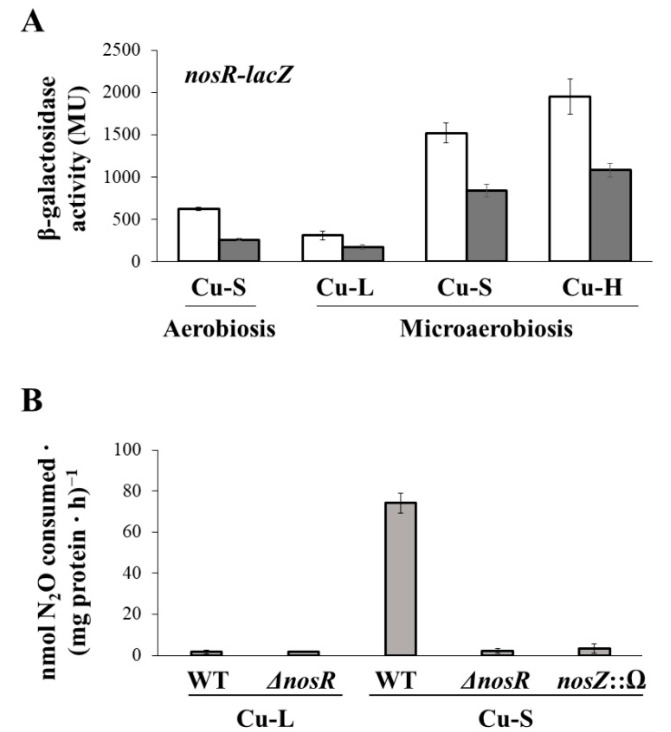
(**A**) β-galactosidase activity from the *nosR-lacZ* transcriptional fusion chromosomally integrated in *B. diazoefficiens* 110*spc*4 (white bars) and *nosR* mutant backgrounds (dark grey bars) grown aerobically in Cu-S or microaerobically in Cu-L, Cu-S and Cu-H BVMN media for 3 days. Data expressed as Miller Units (MU) are means with standard error bars from at least three independent cultures assayed in triplicate. (**B**) Nitrous oxide reductase activity of *B. diazoefficiens* 110*spc*4 (WT) and *ΔnosR* strains incubated in Cu-L and Cu-S BVMN media under microoxic conditions. The *nosZ*::Ω mutant strain cultured in the Cu-S medium was used as negative control in the experiments. N_2_O was measured in the headspace of the cultures after 3 days of incubation. Data represent means with standard error bars from at least two independent cultures assayed in triplicate.

In order to investigate the role of NosR in the expression of the *nos* operon, *B. diazoefficiens* WT and a *nosR* deletion mutant, both containing a fusion between *lacZ* gene and the promoter of the first gene of the *nos* operon, i.e., a *nosR-lacZ* fusion, were grown under microoxic conditions in Cu-L, Cu-S or Cu-H BVMN for 3 days. Expression values obtained under Cu-S aerobic growth were also included as a control. As shown in Figure 7A, β-galactosidase activity of the *nosR-lacZ* fusion was slightly lower in the *nosR* mutant compared with the WT when cells were grown either under oxic (2.4-fold) or under microoxic conditions independently of the concentration of Cu present in the medium (1.8-fold in Cu-L, Cu-S or Cu-H conditions). These results indicate that the lower expression of *nosR-lacZ* in the *nosR* mutant is independent of the growth conditions and could be due to an intrinsic defect of *nos* expression in this mutant. We then investigated N_2_OR activity in the *nosR* mutant compared to the WT, both cultivated microaerobically in Cu-L or Cu-S BVMN media. A *nosZ* mutant strain cultured in the Cu-S medium was employed as a negative control in the experiments. As observed in Figure 7B, N_2_OR activity was very weak in both the WT and the *nosR* mutant when cells were incubated in the Cu-L medium. A significant induction of N_2_OR was observed in the WT grown under Cu-S conditions. However, under these conditions, N_2_OR activity was drastically diminished in the *nosR* mutant compared with the WT (2.03 ± 1.10 versus 74.10 ± 4.89 nmol N_2_O consumed·(mg protein·h)^−1^, respectively). These results indicate that NosR is essential for the optimal function of Nos. 

## 3. Discussion

The main focus of the present work was to contribute to a better understanding of the role of Cu in *B. diazoefficiens* denitrification. To achieve this goal, we undertook an integrated study of the expression of the *nap*, *nirK*, *nor* and *nos* genes that covers gene transcription to expression of the NapA, NirK, NorC and NosZ proteins and the corresponding enzymatic activities involved in the denitrification pathway. Firstly, we analysed the capacity of *B. diazoefficiens* 110*spc*4 to grow under different Cu conditions: Cu limitation (Cu-L), Cu standard (Cu-S) or high Cu (Cu-H). Aerobic growth was not affected by Cu limitation indicating that Cu-independent terminal oxidases can function under these conditions. In fact, like other aerobic facultative bacteria, *B. diazoefficiens* adapts its metabolism to different oxygen conditions through the expression of multiple terminal oxidases with a distinct affinity for oxygen [39]. Eight terminal oxidases have been identified in *B. diazoefficiens*, of which two are Cu-independent *bd*-type oxidases, and the remaining six are heme-Cu oxidases, which use Cu as cofactor [40]. It might be possible that *bd*-type oxidases are responsible for the aerobic growth under Cu limitation. As reviewed by Jünemann (1997) [41], the expression of cytochrome *bd* increased concomitantly with oxygen depletion in *E. coli*; however, major expression levels of this type of cytochrome were observed with the increase in oxygen concentration in *Azotobacter vinelandii*. In the Gram-positive human pathogenic bacterium *Mycobacterium tuberculosis*, Cu-independent cytochrome *bd* oxidases were induced under hypoxia, decreasing Cu requirement as a consequence, which is beneficial for the bacterium because Cu toxicity rises under these conditions [42]. Therefore, an adaptation to oxic Cu-depleted conditions through the synthesis of Cu-independent oxidases in *B. diazoefficiens* would be a plausible hypothesis for the observed phenotype. 

In contrast to oxic conditions, Cu limitation negatively affected anaerobic and microaerobic nitrate-dependent growth. However, other denitrifiers, such as *P. denitrificans* or *A. xylosoxidans* did not show significant growth differences between Cu-L and Cu-H media under anaerobiosis with NO_3_^−^ as the respiratory substrate [34]. The presence of the Cu-independent NirS in *P. denitrificans* could explain the growth differences under Cu-L conditions between this bacterium and *B. diazoefficiens*. However, *A*. *xylosoxidans* that, similarly to *B. diazoefficiens*, possesses a Cu-dependent NirK, was also able to grow anaerobically in a Cu-limiting medium [34]. These different results could be explained by the different growth conditions used in our work and in the aforementioned study, where both *A. xylosoxidans* and *P. denitrificans* were grown as continuous cultures in a chemostat. Another possible explanation for these differences could be given by the fact that both *A. xylosoxidans* and *P. denitrificans* are rapid-growing microorganisms [34], while *B. diazoefficiens* is a slow-growing bacterium. The slow growth rate of *B. diazoefficiens* might have contributed to the negative effect of Cu limitation on the expression and activation of the complete denitrification machinery, and this could be a possible reason for the growth defects observed in Cu-L compared with Cu-S and Cu-H. Contrary to the results observed in this work, Sullivan et al. (2013) [35] did not observe any growth difference between Cu-L and Cu-H in *P. denitrificans* batch cultures. The lack of a growth defect observed in *P. denitrificans* under Cu-L conditions [35] compared with *B. diazoefficiens* could also be explained by the fact that the denitrification process starts by a membrane-bound nitrate reductase (Nar) in *P. denitrificans*, and by a periplasmic nitrate reductase (Nap) in *B. diazoefficiens*. Recent evidence supporting this idea was reported in *P. stutzeri*, another rapid-growing microorganism in which the denitrification process also begins with the reduction of NO_3_^−^ to NO_2_^−^ by a Nar [32]. In the latter study, no significant differences in growth were observed in anaerobic cultures of *P. stutzeri* over a 7-day period throughout a Cu concentration range between 0 and 1 mM Cu. Therefore, our work provides evidence that Cu limitation could affect growth of slow-growing microorganisms provided with both a Nap as the first enzyme of the denitrification process and a Cu-dependent NirK. 

To better understand the effect of Cu on *B. diazoefficiens* denitrification, we investigated gene and protein expression, as well as the activity of each denitrification enzyme in microaerobic Cu-L, Cu-S and Cu-H cultures. By using transcriptional fusions to the reporter gene *lacZ*, we demonstrated the Cu dependence of *nirK*, *nor* and *nos* denitrification gene induction under microoxic conditions. These observations were confirmed by performing qRT-PCR analyses of the *napA*, *nirK*, *norC* and *nosR* genes. Similarly to our observations, adequate bioavailable Cu concentrations (0.15 mM) resulted in the greatest transcription levels of *P. stutzeri nirS*, *norB* and *nosZ* denitrification genes compared with low Cu [32]. Moreover, low levels of *nosZ* expression during Cu limitation were also reported in *P. denitrificans* [35]. Interestingly, in addition to *nirK* and *nos* genes encoding the Cu-dependent NirK and Nos, induction of expression of *nor* genes, which encode the Cu-independent Nor, also requires Cu. We demonstrated that this control is not mediated by the low expression levels of *nirK*, which possibly decrease NO production, the signal molecule required to induce *nor* gene expression [25]. Thus, we propose a negative effect of Cu limitation at the *nor* gene transcriptional level. 

Gene expression results clearly indicated that the induction of *nirK*, *nor* and *nos* genes by low oxygen and nitrate was significantly reduced under Cu limitation, suggesting that this control might be modulated by a specific repressor. In this context, CsoR and CopY are considered as Cu-sensing transcriptional ATPase repressors in bacteria, such as *M. tuberculosis* [43], *E. coli* and *Enterococcus hirae* (for a review, see Rademacher and Masepohl, 2012 [44]). Nevertheless, a *csoR-cueA* divergon encoding a CsoR-like repressor and a heavy metal transporting P-type ATPase (CueA) was recently reported in the Gram-negative bacterium *Bradyrhizobium liaoningense* CCNWSX0360 [45]. These authors attributed a crucial role in Cu homeostasis to this system, as well as in zinc/cadmium resistance. A search of the *B. diazoefficiens* genome in the KEGG database (https://www.genome.jp/kegg/, accessed on 29 July 2020) revealed the presence of a putative gene encoding a repressor from the CsoR family whose annotation is *bsr0701*. Apart from the probable role of this repressor in metal tolerance, a putative role of the predicted CsoR protein in the low transcription levels of *B. diazoefficiens nirK, nor* and *nos* under Cu limitation requires further investigation. 

The lack of response of *nap* genes to Cu was corroborated by analysing NapA levels which, similarly to gene expression results, were not affected by the Cu concentration present in the medium. In contrast, a clear effect of Cu on NR activity was observed. This effect was variable through the incubation period. While NR activity was higher after 1 day of incubation under Cu-L conditions compared with Cu-S, it was notably inhibited after 2 or 3-day incubation periods. Affinities for metals generally follow a universal order of preference, which for essential divalent metals is the Irving-Williams series: Zn^2+^ < Cu^+^ > Cu^2+^ > Ni^2+^ > Co^2+^ > Fe^2+^ > Mn^2+^ > Mg^2+^ > Ca^2+^ [46]. Normally, each metal ion can replace another metal ion downstream that series. Thus, cupric ion (Cu^2+^) is highly competitive and is expected to substitute metal cofactors of different metalloproteins, especially those containing sulfur and nitrogen ligands. NapA contains an Mo[MGD]_2_ cofactor and a [4Fe-4S] centre [14,19]. Therefore, it might be possible that a competition for the active sites of NapA may exist between Cu and Fe or Mo [46]. Under Cu limitation, the low concentration of Cu^2+^ ions would not be able to compete with Fe or Mo, resulting in major NR activity. The inhibition of Nap activity observed after 2 and 3 days of incubation under Cu-L conditions might be due to the high levels of nitrite present in the medium from the first day. Nitrite accumulation might also explain the observed growth defect under Cu-L conditions similarly, as has been previously observed for a *nirK* mutant that also accumulates nitrite in the medium [20,38]. Contrary to the response to Cu limitation, NR activity was induced after 2 or 3 days of incubation under Cu-H conditions. Interestingly, the NR response to Cu-L, Cu-S and Cu-H clearly correlated with nitrate consumption rates. Taken together, these results suggest a post-translational control of *B. diazoefficiens* Nap by Cu. 

With regard to NirK, and likewise *nirK* gene expression, protein levels decreased under Cu-L conditions compared with Cu-S. However, this effect was more pronounced in the periplasm than in the cytosolic fraction. In contrast, in Cu-H grown cells, NirK was expressed more in the periplasm than in the cytoplasm. The sequence of *B. diazoefficiens* NirK shows the sequence ^3^TRRAALI^9^ in the N-terminal region [20] that corresponds closely to the `twin arginine’ motif (S/T)RRXFLK identified in a large number of periplasmic metalloproteins that contain complex cofactors and are exported via the Sec-independent Tat system [47,48]. In fact, Cu-NirK exportation via the Tat system was demonstrated in some halophilic Archaea, such as *Haloferax mediterranei* [49]. Our results suggest that, in addition to influencing *nirK* expression, Cu might also have an effect on NirK transport to the periplasm. The low levels of NirK in response to Cu limitation explain the low NIR activity, as well as the high levels of nitrite accumulation. However, although Cu-H did not increase NirK content in the periplasm compared with Cu-S, a major activation of NirK activity occurred in Cu-H-grown cells, which could explain the absence of nitrite in the Cu-H growth medium. These results suggest a post-translational effect of high Cu on NirK catalytic activity. In this context, it was recently reported that pH causes structural changes in *B. diazoefficiens* USDA 110 NirK, resulting in a pH-dependent catalytic activity [50]. 

The negative effect of Cu limitation in *nor* gene expression resulted in low levels of NorC protein, as well as Nor activity. In spite of the lack of induction of *nor* gene expression by Cu-H compared with Cu-S, a significant increase in NorC levels, as well as Nor activity, was observed in the former. These results suggest a post-transcriptional control by Cu-H on NorC synthesis. Since Cu-H significantly increases NirK activity and consequently NO formation, it might be possible that cells need to produce higher cNor levels to protect themselves from NO toxicity under these Cu conditions.

Regarding Nos, there is a transcriptional control of *nos* gene expression under Cu-L conditions. In contrast, Cu-H did not significantly influence *nos* gene expression compared with Cu-S, but it did so on the levels of NosZ, as well as N_2_OR activity, highlighting the relevance of Cu for the proper maturation and function of NosZ. The higher levels of the mature NosZ, compared with those of the truncated NosZ observed in the periplasm of Cu-H-grown cells, suggest that the post-transcriptional regulation of Nos by Cu-H probably affects the maturation of the protein in the periplasm. In this context, it was reported that, despite being transported by the Tat system, NosZ maturation is completed in the periplasm where Cu_A_ and Cu_Z_ centres are assembled to the NosZ apoprotein [14]. Our results are consistent with previous observations in other microorganisms [33,34,51], where N_2_OR activity was modulated by Cu. In our work, the low N_2_OR activity in Cu-L conditions provoked N_2_O accumulation in the headspace. Similarly, in *P. denitrificans* Cu-L batch cultures with nitrate, Sullivan et al. (2013) [35] observed a transient accumulation of N_2_O that was not observed in the Cu-H culture, suggesting that the catalytic capacity of the other denitrification reactions exceeded the rate of the Cu-dependent Nos. 

Taking the above-mentioned results together, we conclude that activation of Nap, NirK, Nor and Nos activity under Cu-H (13 µM) conditions could explain the greater capacity to grow, compared with that observed in the Cu-S medium (0.02 µM). While Cu limitation clearly inhibits *B. diazoefficiens* denitrification, 13 µM Cu induces growth, as well as the activity of the denitrification enzymes. 

The biological reduction of N_2_O to N_2_, which is the last step of the denitrification process, is catalysed by the Nos enzyme, encoded by the *nosRZDFYLX* operon in *B. diazoefficiens* [22]. In general, NosZ is the catalytic subunit, encoded by the *nosZ* gene. NosZ is a periplasmic homodimeric protein that contains two copper centres, Cu_A_ and Cu_Z_, in each monomer [52]. NosR is a polytopic integral membrane protein that serves as electron donor for N_2_O reduction [53]. This protein possesses a periplasmic FMN-binding domain and a C-terminal ferredoxin-like domain with two [4Fe-4S] clusters located in the cytoplasm [54]. It has been suggested that NosR and a member from the ApbE protein family, NosX, are involved together in N_2_O reduction in vivo maintaining the catalytic activity of NosZ. Following this line, the existence of an electron-donor system via NosR was generally proposed, in which NosX would perform as a quinol oxidoreductase, which could be parallel to that involving cytochrome *bc*_1_, cytochrome *c*_550_ and pseudoazurin [53,55]. Recently, Zhang et al. (2017) [56] showed that the periplasmic FAD-binding protein ApbE from *P. stutzeri* catalyses the flavinylation of the FMN-binding domain of NosR. This evidence strongly suggests the role of ApbE (or NosX in other microorganisms, such as *P. denitrificans* or *B. diazoefficiens*) in sustaining the catalytic activity of NosZ via NosR. In the present work, we provide evidence that NosR would be required for Nos activity in *B. diazoefficiens*, confirming previous results from our group [22]. Nevertheless, further investigation is required to elucidate the participation of NosX in Nos activity in *B. diazoefficiens*. In addition to its proposed role as electron donor to NosZ, a regulatory role for NosR has also been suggested, since Honisch and Zumft (2003) [57] reported that NosR was required for the transcription of *nosZ* and *nosD* in *P. stutzeri*. Experimental evidence provided by Wunsch and Zumft (2005) [53] suggested an indirect control of NosR on its target genes and that only the periplasmic flavin-containing domain was needed for *nosZ* expression. Additionally, Sullivan et al. (2013) [35] analysed the transcription of *nosZ* in *P. denitrificans* parental or *nosR* mutant backgrounds under anoxic Cu-L conditions with nitrate. They found that transcript levels in *nosR* mutant were notably higher compared with those in the wild-type strain, providing strong evidence of a role for NosR in the repression of *nosZ* expression in response to Cu limitation in *P. denitrificans*. Conversely, the present work provides evidence that NosR would not be involved in the expression of the *nosRZDFYLX* operon in *B. diazoefficiens* in response to microoxia or Cu. Contrary to our results, Velasco et al. (2004) [22] reported very low levels of β-galactosidase activity in cells of a *nosR* mutant that contains a *nosZ-lacZ* fusion incubated microaerobically in a complete medium (YEM), leading those authors to suggest a possible role of NosR as a positive regulator of the *nosZ* gene, as postulated by Zumft (1997) [8]. In fact, the transcription of *nosRZDFYLX* mainly depends on a promoter present in the DNA region upstream of *nosR* as previously reported [26]. However, β-galactosidase activity from a *nosZ*-*lacZ* fusion was also observed [26], which cannot exclude the possibility that another internal promoter upstream of *nosZ* might exist. An analysis of *nosZ* expression in a *nosR* mutant background under Cu limitation would help to clarify this discrepancy. 

## 4. Materials and Methods

### 4.1. Bacterial Strains and Growth Conditions 

Bacterial strains used in this study are compiled in Table 1. *Bradyrhizobium diazoefficiens* cells were cultivated routinely under oxic conditions at 30 °C in peptone–salts–yeast extract (PSY) medium supplemented with 0.1% L-arabinose, essentially as described by Mesa et al. (2008) [24]. Buffered Vincent’s minimal medium, here defined as vitamin-free modified Vincent’s minimal medium (BVM, [58,59]) was used in this study, containing the following ingredients (per litre): KH_2_PO_4_, 2 g; K_2_HPO_4_, 2 g; NH_4_Cl, 840 mg; MgSO_4_·7H_2_O, 246.48 mg; CaCl_2_·2H_2_O, 67.63 mg; FeCl_3_·6H_2_O, 10 mg; MOPS, 2.09 g. This medium was supplemented with 3 g of 1 M arabinose and 1 mL from a mineral solution [60] consisting of; H_3_BO_3_, 145 mg; ZnSO_4_·7H_2_O, 108 mg; Na_2_MoO_4_·2H_2_O, 125 mg; MnCl_2_·4H_2_O, 4 mg; FeSO_4_·7H_2_O, 125 mg; CoSO_4_·7H_2_O, 70 mg; nitrile triacetate, 7 g; CuSO_4_·5H_2_O, 5 mg. When needed, the medium was supplemented with 10 mM KNO_3_ (referred here as BVMN). Final pH was adjusted around 6.8 with 2 M NH_3_. 

Final Cu concentration in BVM or BVMN as indicated in the original recipe [58] was 0.02 µM, referred to in this manuscript as Cu-standard medium (Cu-S). In this study, 13 µM Cu was used as high Cu conditions (Cu-H); this concentration was also used as Cu-H in previous studies [34,35]. In the case of the Cu-limiting medium (Cu-L), CuSO_4_·5H_2_O was omitted from the mineral solution, and 10 µM bathocuproine disulfonic acid (BCS) (Cu(I) chelator) and 1 mM L-ascorbate (reducer from Cu(II) to Cu(I)) were added to the medium in order to lower Cu availability [34,36]. Only for the Cu-L medium, glassware was treated overnight with 0.1 M HCl and rinsed afterwards with double-distilled water [36]. 

After growing under oxic conditions in the PSY medium, *B. diazoefficiens* cells were collected by centrifugation (8000× *g*, 8 min, 4 °C). Next, cells were washed twice with BVM or BVMN and inoculated at an OD_600_ of 0.05 (or 0.2 when needed). For oxic conditions, 3 mL of medium were added to 17-mL tubes. For anoxic conditions, 17-mL tubes were completely filled with medium. For microoxic conditions, 3, 50 and 100 mL of medium were added to 17-mL, 250 and 500-mL rubber stoppered tubes or Erlenmeyer flasks, respectively. The headspace was then filled with a gas mixture consisting of 2% (*v/v*) O_2_ and 98% (*v/v*) N_2_ and both, tubes and flasks, were incubated at 30 °C with agitation at 170 rpm.

When needed, antibiotics were added to *B. diazoefficiens* cultures at the following concentrations (µg mL^−1^): spectinomycin (Spc), 200 (solid cultures), 100 (liquid cultures); streptomycin (Sm), 200 (solid cultures), 100 (liquid cultures); tetracycline (Tc), 100 (solid cultures), 25 (liquid cultures); kanamycin (Km), 200 (solid cultures), 100 (liquid cultures); chloramphenicol (Cm), 20. 

### 4.2. Analysis of Gene Expression by qRT-PCR

Expression of *napE*, *nirK*, *norC* and *nosR* was analysed by qRT-PCR using a QuantStudio 3 Real-Time PCR system (Thermo Fisher Scientific, Waltham, MA, USA). *B. diazoefficiens* 110*spc*4 was grown microaerobically in the BVMN medium under Cu-L or Cu-S conditions for 48 h. Cell harvest, isolation of total RNA and cDNA synthesis were performed as described previously [24,63,64]. Primers for the PCR reactions (Table S1) were designed with the Clone Manager Suite software to have melting temperatures between 57 and 62 °C and generate PCR products of 75–100 bp. Each PCR reaction contained 9.5 µL of iQTM SYBR Green Supermix (Bio-Rad, Hercules, CA, USA), 2 mM (final concentration) of individual primers and appropriate dilutions of different cDNA samples in a total volume of 19 µL. Reactions were run in triplicate. Melting curves were generated to verify the specificity of the amplification. Relative changes in gene expression were calculated as described by Pfaffl (2001) [65]. Expression of the 16S *rrn* gene was used as reference for normalization (Table S1). 

### 4.3. Analytical Methods 

The nitrite concentration accumulated in the growth medium was estimated colorimetrically after diazotisation by adding the sulphanilamide/naphtylethylene diamino dihydrochloride reagent [66] and extrapolating from a standard curve constructed with increasing concentrations of NaNO_2_ from a stock solution of 100 µM: 0, 20, 40, 60, 80 and 100 µM. The nitrate concentration present in the growth medium was also estimated colorimetrically using the Nitrite/Nitrate Assay Kit (Sigma-Aldrich, Saint Louis, MO, USA), based on the method of Miranda et al. (2001) [67]. 

Protein concentration was estimated colorimetrically after alkaline lysis (1 N NaOH at 100 °C for 20 min) using the Bradford reagent (Bio-Rad) and extrapolating from a standard curve constructed with increasing concentrations of bovine serum albumin (BSA) from a stock solution of 100 µg mL^−1^: 0, 4, 8, 12, 16 and 20 µg mL^−1^ [68]. 

### 4.4. Determination of β-Galactosidase Activity 

β-galactosidase activity was analysed using permeabilised cells from at least three independent cultures (3 mL), assayed in triplicate for each strain and condition, as previously described [69]. Specific activity was calculated in Miller Units [70]. To analyze β-galactosidase activity in the presence of NO, this gas was generated chemically according to Bricio et al. (2014) [71] and added (50 µM final concentration) to the tubes 5 h before activity measurements. 

### 4.5. Determination of N_2_O Production 

*B. diazoefficiens* cells were cultured in serum tubes under microoxic conditions as indicated above. Cultures were incubated at 30 °C and 170 rpm for 7 days. N_2_O was measured using a Hewlett Packard HP-4890D gas chromatography instrument equipped with an electron capture detector (ECD) (Hewlett Packard, San Jose, CA, USA), as essentially described by Torres et al. (2014) [72]. The column was packed with Porapak Q 80/100 mesh. N_2_ was used as the carrier gas circulating at a flow rate of 23 mL min^−1^. The injector, column and detector temperatures were 125, 60 and 375 °C, respectively. Gas samples were taken from the headspace of the cultures after 1, 2, 3, 6 and 7 days and injected manually using luer-lock gas-tight syringes BD Microlance^TM^ 3. Peaks corresponding to N_2_O were integrated using GC ChemStation Software (Agilent Technologies, Santa Clara, CA, USA) and the values obtained were used to calculate the N_2_O concentration in each sample through extrapolation from a standard curve, performed using 2% (*v/v*) N_2_O standard (Air Liquid, Paris, France) and including the following gas volumes: 0, 0.2, 0.4, 0.6, 0.8 and 1 mL. Total N_2_O concentration was determined taking into account N_2_O in headspace and dissolved N_2_O, applying the Bunsen solubility coefficient (47.2% at 30 °C). 

### 4.6. Determination of Nitrate (NR, EC 1.7.99.4) and Nitrite Reductase (NIR, EC 1.7.2.1) Activities 

Three sets of 250-mL flasks were inoculated at an initial OD_600_ of 0.05 (or 0.2 when *nirK* mutant was included) in BVMN and incubated at 30 °C and 170 rpm under microoxic conditions for 1, 2 and 3 days, respectively, for each set. After each incubation time, cells were collected by centrifugation at 8000× *g* for 8 min at 4 °C, washed 4–5 times with 50 mM Tris-HCl buffer (pH 7.5) to remove all the possible NO_2_^−^ accumulated in the medium, and then resuspended in 0.5–1 mL of the same buffer. Methyl viologen-dependent nitrate reductase (MV^+^-NR) and nitrite reductase (MV^+^-NIR) activity measurements were performed as essentially described by Delgado et al. (2003) [19]. The reaction mixtures contained 200 µM methyl viologen (MV^+^), 0.1–0.2 mg of protein from the cell suspension, 50 µL distilled water, and 10 mM KNO_3_ or 100 µM NaNO_2_ for MV^+^-NR or MV^+^-NIR activity, respectively, adding 50 mM Tris-HCl buffer to reach a final volume of 450 µL in each reaction tube. Before measurements, 46 mM sodium dithionite solution was freshly prepared (8 mg mL^−1^ in 50 mM Tris-HCl buffer [pH 7.5]). Next, 50 µL of that solution was added to each reaction tube. After incubation for 10–20 min at 30 °C, the reaction was stopped by vigorous shaking until the blue colour disappeared from the samples. Controls were prepared by vigorously shaking immediately after the addition of dithionite. MV^+^-NR and MV^+^-NIR activities were expressed as nmol NO_2_^−^ produced or consumed·(mg protein·min)^−1^. Two biological replicates for each Cu condition were used. 

### 4.7. Determination of Nitric Oxide Reductase (NOR, EC 1.7.2.5) Activity 

A set of 500-mL flasks was inoculated at an initial OD_600_ of 0.05 in BVMN and incubated at 30 °C and 170 rpm under microoxic conditions for 3 days. Cells were then harvested and washed in a similar way as for the MV^+^-NR and MV^+^-NIR activity assays and resuspended in 1.5 mL of the same buffer. NO consumption rates were determined using a 2-mm ISONOP NO electrode APOLLO 4000^®^ (World Precision Instruments, Friedberg, Germany), following Cabrera et al. (2016) [69]. The reaction chamber (≈2.2 mL) contained 1.8 mL phosphate buffer 25 mM (pH 7.41), 100 µL of cell suspension (0.3–0.5 mg protein), 90 µL of 1 M sodium succinate, 100 µL of 320 mM glucose, and 100 µL of an enzyme mix containing 40 units mL^−1^ of *Aspergillus niger* glucose oxidase and 250 units mL^−1^ of bovine liver catalase. Once a steady base line was obtained, 50 µL of a saturated NO solution of 1.91 mM (at 20 °C) [71] was added to the chamber to start the reaction. Each measurement was stopped when the NO concentration had dropped to zero, meaning that all the NO present had been consumed. NOR activity was expressed as mmol NO consumed·(mg protein·h)^−1^. Two biological replicates for each Cu condition were used. 

### 4.8. Determination of Nitrous Oxide Reductase (N_2_OR, EC 1.7.2.4) Activity 

Three sets of 250-mL flasks were inoculated at an initial OD_600_ of 0.05 in BVMN and incubated at 30 °C and 170 rpm under microoxic conditions for 3 days. Cells were then harvested, washed in a similar way as for the MV^+^-NR and MV^+^-NIR activity assays, and resuspended in 1.5 mL of the same buffer. N_2_O consumption rates were measured as essentially described by Jiménez-Leiva et al. (2019) [27]. The assay was performed in 17-mL serum tubes capped with rubber septa containing 6.3 mL of 50 mM Tris-HCl buffer (pH 7.5) and 700 µL of 600 mM sodium succinate as the electron donor. Before cells were added, the tubes were supplied with a mixture of 2% (*v/v*) O_2_ and 98% (*v/v*) N_2_ and then, 500 µL of 2% (*v/v*) N_2_O from a gas mixture with 98% (*v/v*) N_2_ were injected in each tube. Next, all the tubes were incubated at 30 °C with agitation for 30 min to ensure equilibrium between gas and liquid phases. After this incubation, a volume of cell suspension corresponding to 0.3–0.4 mg protein was injected manually into each reaction tube. When reaction tubes were set up, 1-mL aliquots were taken from the headspace of each tube at the start of the reaction and four hours after incubation at 30 °C. N_2_O measurements and concentration calculations were performed as described in Section 4.5. N_2_OR activity was expressed as nmol N_2_O consumed·(mg protein·h)^−1^. Two biological replicates for each Cu condition were used. 

### 4.9. Detection of Membrane-Bound NorC by Heme-Staining 

A set of 500-mL flasks was inoculated at an initial OD_600_ of 0.05 in BVMN and incubated under microoxic conditions at 30 °C and 170 rpm for 3 days. Cells were then collected at 8000× *g* for 8 min at 4 °C. After sedimentation, cells were washed once with a solution of 150 mM KCl in Tris-HCl 50 mM (washing buffer, pH 7). Washed cells were finally resuspended in 1.5 mL of fractionation buffer, consisting of wash buffer containing 1 mM of 4-(2-aminoethyl) benzenesulfonyl fluoride hydrochloride (AEBSF). Cell fractionation, membrane isolation, sodium dodecyl sulphate gel electrophoresis (SDS-PAGE) and heme-staining were performed as essentially described by Delgado et al. (2003) [19]. Cells were disrupted up to three times using a cold French pressure cell (SLM Aminco, MD, USA) at about 120 MPa, and subsequently centrifuged at 20,000× *g* for 10 min at 4 °C to remove cell debris. These extracts were immediately centrifuged at 140,000× *g* for 45 min at 4 °C using a tabletop ultracentrifuge Optima Max (Beckman Coulter Inc., Brea, CA, USA). Cell membranes were resuspended in fractionation buffer and a volume equivalent to 30 µg of protein was mixed with freshly prepared SDS loading dye 6x (350 mM Tris-HCl, pH 6.8, 30% glycerol, 20 mM DTT, 350 mM SDS, 0.05% bromophenol blue), adding distilled water up to a final volume of 12 µL. Samples prepared were consecutively loaded without boiling onto 4%-stacking and 14%-resolving-SDS-PAGE polyacrylamide gels, resolved and transferred to a nitrocellulose membrane using Trans-Blot Turbo System (Bio-Rad). Heme-dependent peroxidase activity was detected using the Amersham^TM^ ECL Select^TM^ Western Blotting Detection Reagent Kit (GE Healthcare, Chicago, IL, USA) and chemiluminescence signals were revealed with a ChemiDoc XRS+ System. Images were analysed operating with the Quantity One and Image Lab^TM^ softwares (Bio-Rad). Two biological replicates for each Cu condition were analysed. 

### 4.10. Detection of NapA, NirK and NosZ by Immunoblot Analyses 

A set of 500-mL flasks was inoculated with *B. diazoefficiens* 110*spc*4 at an initial OD_600_ of 0.05 in BVMN and incubated under microoxic conditions at 30 °C and 170 rpm for 3 days. Additionally, *napA*, *nirK* and *nosZ* mutant strains were used as the negative control in the experiments, being inoculated at an initial OD_600_ of 0.2, 0.2 and 0.05, respectively, in order to collect a sufficient cell mass of each strain. Cells were then harvested at 3740× *g* for 15 min at 4 °C. Periplasmic proteins were isolated following a protocol modified from Felgate et al. (2012) [34]. Cells were resuspended in 10 mL of SET buffer, containing 500 mM sucrose, 100 mM Tris-HCl and 3 mM EDTA at pH 8. Next, 1 mg mL^−1^ lysozyme was added and the mix was incubated at 30 °C for 2 h. Subsequently, cells were centrifuged at 12,000× *g* for 15 min to separate periplasmic proteins from spheroplasts. The periplasmic fraction was then consecutively concentrated up to 4 mL and 250 µL using Amicon^®^ Ultra tubes of 3 kDa and 10 kDa molecular weight cut-off (MWCO), respectively. After centrifugation of the tubes at 7500× *g* for 20 min, periplasmic proteins were stored at −20 °C until use. The subsequent spheroplasts fractionation was performed as described in Section 4.9. After ultracentrifugation, cytosolic fractions were collected. For SDS-PAGE, a volume equivalent to 21 or 14 µg of periplasmic or cytosolic proteins, respectively, was then mixed with freshly prepared SDS loading dye 6x, and distilled water added up to a final volume of 20 or 12 µL, respectively. Samples were heated at 95 °C for 10 min, centrifuged at 26,000× *g* for 5 min, and were then loaded onto 4%-stacking and 14%-resolving-SDS-PAGE polyacrylamide gels, resolved and transferred to a nitrocellulose membrane using Trans-Blot Turbo System (Bio-Rad). NapA immunoblot analyses were performed according to Delgado et al., 2003 [19] using antibodies raised against purified *P. pantotrophus* NapA at a dilution rate of 1:750 [37]. NirK immunoblotting was performed using a homologous rabbit primary antibody of *B. diazoefficiens* (ABclonal Biotechnology Co., Wuhan, China) at a dilution rate of 1:1000, while the secondary antibody utilised was a horseradish peroxidase (HRP)-conjugated anti-rabbit one (Sigma-Aldrich) at a dilution rate of 1:3500. NosZ immunoblotting was carried out as previously described [26,27,34], using a heterologous sheep primary antibody of *P. denitrificans* at a dilution rate of 1:1000 together with a HRP-donkey anti-sheep secondary antibody (Sigma-Aldrich) at a dilution rate of 1:3500. Detection of peroxidase activity and image processing were performed as described in Section 4.9. 

### 4.11. Statistical Analysis 

The total number of replicates is given in each figure. Data were checked for normal distribution according to Kolmogorov–Smirnov and Shapiro–Wilk tests. We then performed inferential statistics to test null hypotheses applying a parametric ANOVA for unpaired treatments. Next, a *post-hoc* Tukey HSD test at *p* ≤ 0.05 with SPSS software was performed. 

## 5. Conclusions

The main goal of the present work was to investigate the influence of Cu on denitrification in the soybean endosymbiont *B. diazoefficiens*. Taken together, our results suggest that Cu not only affects enzymatic activity of Nap, NirK, cNor or Nos enzymes, but also may act as an essential factor in the regulation of the denitrification gene expression, as well as mediate the transport and maturation of the Cu-dependent NirK and NosZ enzymes, respectively. Therefore, Cu could be involved in the denitrification regulatory network and not only acts as a mere enzymatic cofactor of the Cu-dependent enzymes, but also as an important regulatory signal of this process. 

## Figures and Tables

**Table 1:** *B. diazoefficiens* strains used in this study.

Strains	Relevant Description	Source of Reference
110*spc*4	Cm^r^ Spc^r^ wild-type, a spectinomycin resistant derivative of USDA110	[61]
GRPA1	Cm^r^ Spc^r^ Sm^r^ *napA*::Ω	[19]
GRK308	Cm^r^ Spc^r^ Sm^r^ *nirK*::Ω	[20]
GRC131	Cm^r^ Km^r^ *norC::aphII*-PSP	[21]
GRZ3035	Cm^r^ Spc^r^ Sm^r^ *nosZ*::Ω	[22]
*Δ* *nosR*	Cm^r^ Spc^r^ *B. diazoefficiens* 110*spc*4 markerless deletion mutant	Laboratory collection
BG0602	Cm^r^ Tc^r^ *napE-lacZ* chromosomally integrated into USDA110	[62]
RJ2498	Cm^r^ Spc^r^ Tc^r^ *nirK-lacZ* chromosomally integrated into 110*spc*4	[23]
RJ2499	Cm^r^ Spc^r^ Tc^r^ *norC-lacZ* chromosomally integrated into 110*spc*4	[23]
BG0301	Cm^r^ Spc^r^ Tc^r^ *nosR-lacZ* chromosomally integrated into 110*spc*4	[26]
*ΔnosR*-BG0301	Cm^r^ Spc^r^ Tc^r^ *nosR-lacZ* chromosomally integrated into Δ*nosR*	Laboratory collection

## Data Availability

Not applicable.

## Supplementary Materials

The following are available online at https://www.mdpi.com/article/10.3390/ijms23063386/s1.
