# Supplementary material for: Effect of Copper on Expression of Functional Genes and Proteins Associated with Bradyrhizobium diazoefficiens Denitrification
Source: Int J Mol Sci. 2022 Mar 21;23(6):3386. doi: 10.3390/ijms23063386 (PMC8951191; doi:10.3390/ijms23063386)
Supplement: Supplementary file 1 [file ijms-23-03386-s001.zip › Supplementary Figures and Table.pdf]

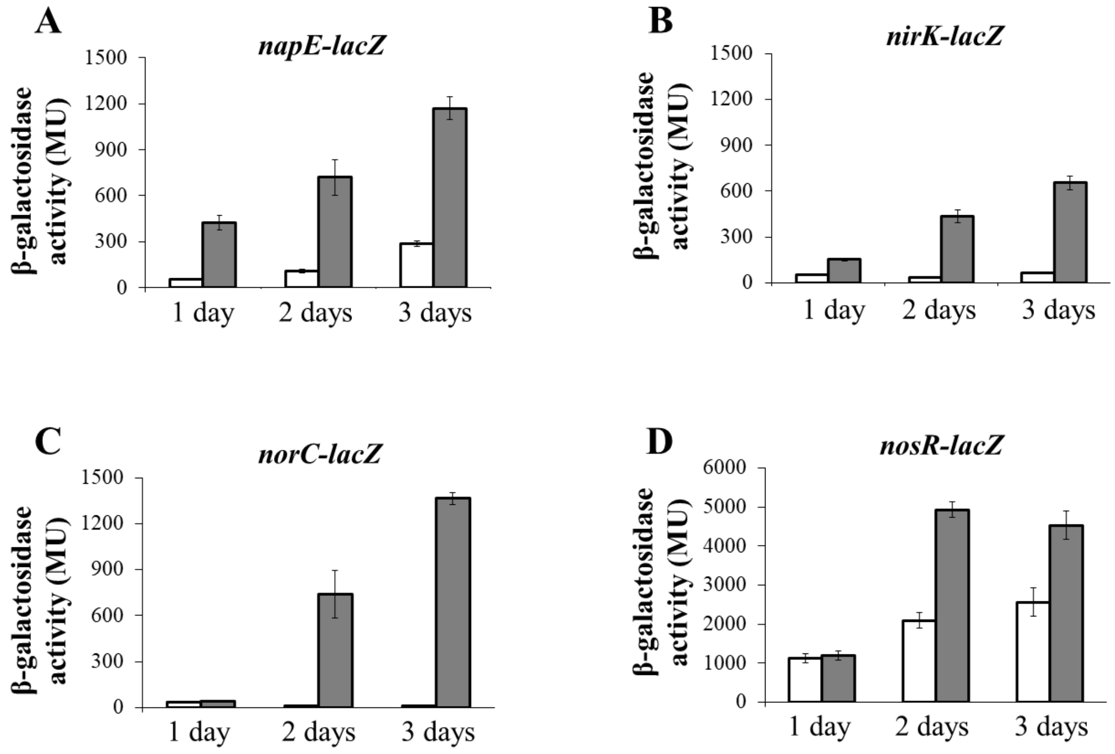

Figure S1. β-galactosidase activity from *napE-lacZ* **(A)**, *nirK-lacZ* **(B)**, *norC-lacZ* **(C)** and *nosR-lacZ* **(D)** transcriptional fusions chromosomally integrated in *B. diazoefficiens* 110*spc*4 grown under aerobic (white bars) or microaerobic conditions (dark grey bars) in Cu-S BVMN medium for 1, 2 and 3 days. Data expressed as Miller Units (MU) are means with standard error bars from at least three independent cultures assayed in triplicate.

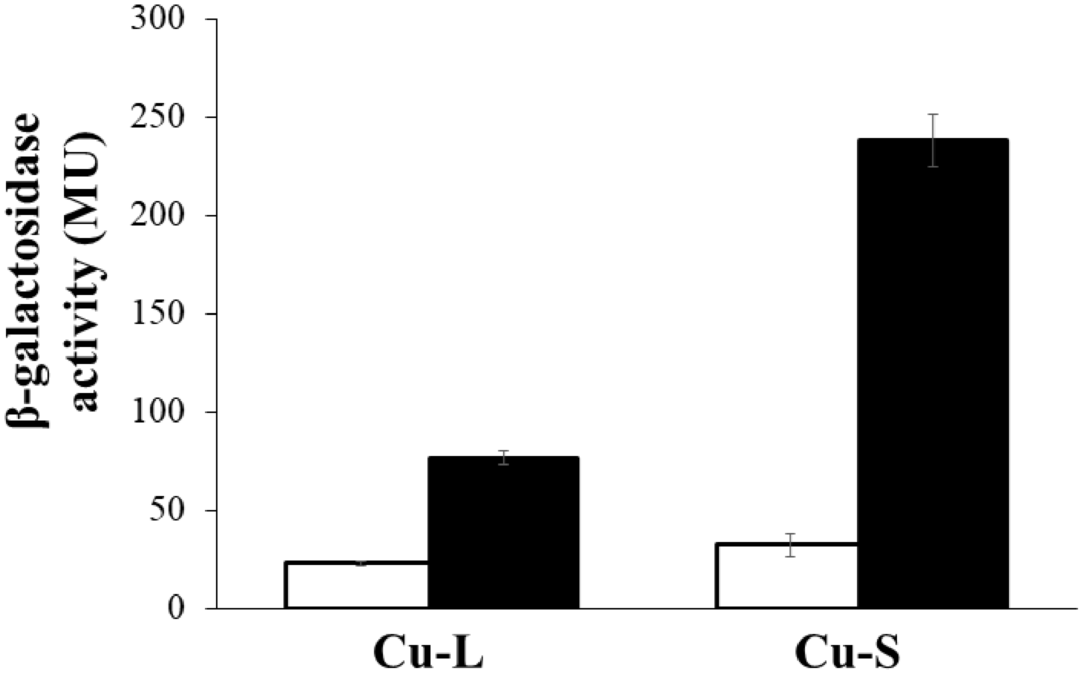

Figure S2. β-galactosidase activity from the *norC-lacZ* transcriptional fusion chromosomally integrated in *B. diazoefficiens* 110*spc*4 grown under microaerobic conditions in Cu-L or Cu-S BVM medium (*i. e.* without nitrate). Tubes were incubated during 24 h at an initial OD_{600} of 0.2, and 5 h before the assay, NO was added (black bars) or not (white bars). Data expressed as Miller Units (MU) represent means with standard error bars from at least three independent cultures assayed in triplicate.

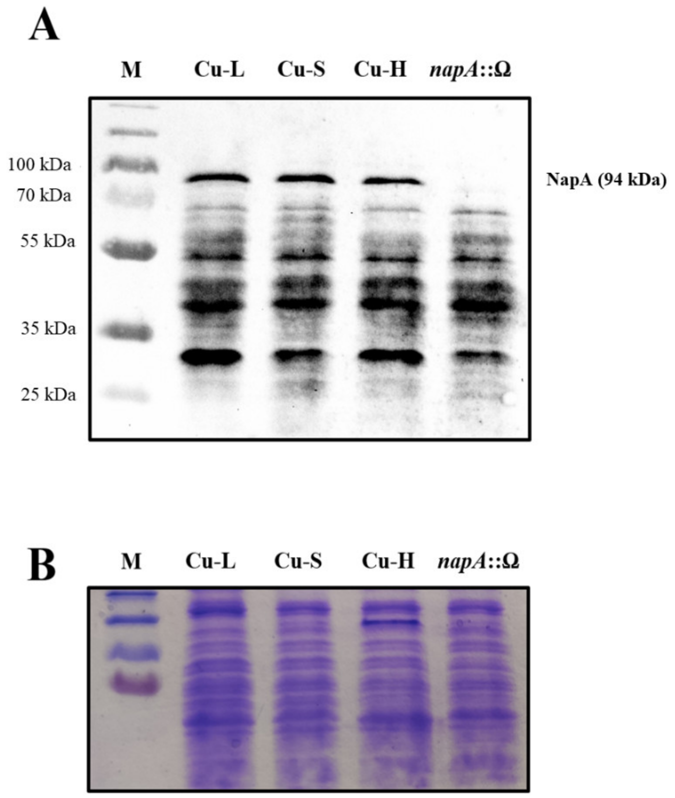

Figure S3. (A) Full scans of the entire gel of the western blot experiments shown in Figure 3A. Apparent masses of the marker proteins (kDa) are shown at the left margin. **(B)** Coomasie® blue-stained SDS-PAGE gels made to visualize protein levels loaded in each lane for NapA western blot experiments. M, protein marker ladder.

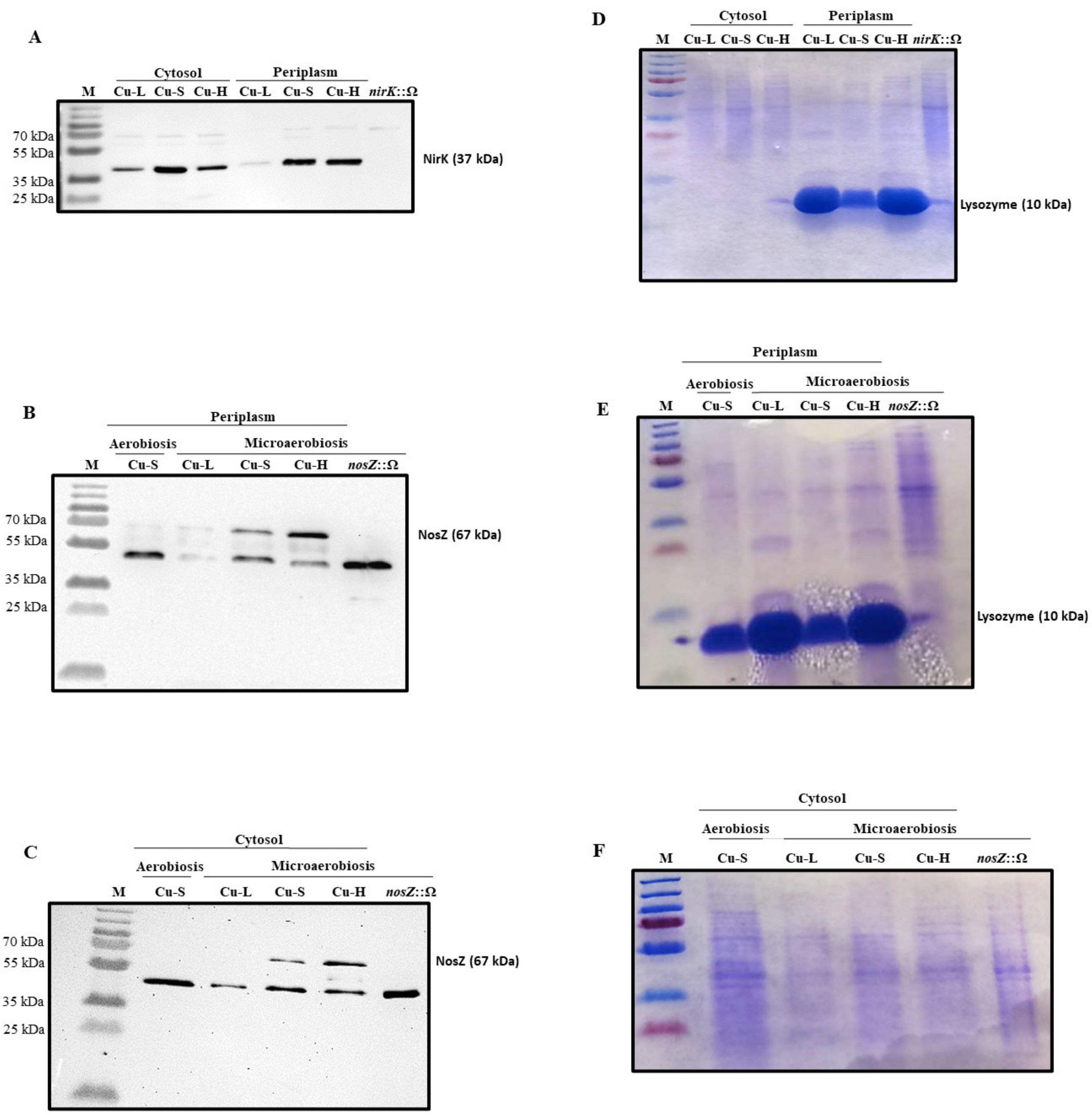

Figure S4. **(A,B,C)** Full scans of the entire gels of the western blot experiments shown in Figure 4A **(A)** and 6A **(B,C)**. Apparent masses of the marker proteins (kDa) are shown at the left margin. **(D,E,F)** Coomasie® blue-stained SDS-PAGE gels made to visualize protein levels loaded in each lane for NirK **(D)** and periplasmic **(E)** or cytosolic **(F)** NosZ western blot experiments. M, protein marker ladder.

A

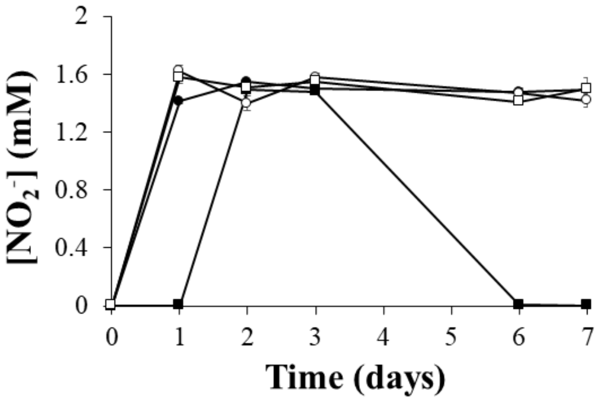

B

	MV⁺-NR	
	Microaerobic conditions + 10 mM KNO_3	
	**Cu-L**	**Cu-S**
**WT**	0.30(0.02)	22.41(0.70)
***nirK*::Ω**	0.45(0.10)	0.84(0.09)

Figure S5. (A) Extracellular nitrite concentration in the growth medium of *B. diazoefficiens* 110*spc*4 (WT, black symbols) and the *nirK*::Ω mutant strain (open symbols) cultured under Cu-L (●, ○) or Cu-S (■, □) conditions. Error bars represent standard error between triplicates, and where not visible, these were smaller than the symbols. **(B)** Methyl viologen-dependent nitrate reductase (MV^+-NR) activity of WT and *nirK*::Ω mutant strains cultured in Cu-L or Cu-S. MV^+-NR activity given as nmol NO_2^- produced x (mg protein min)$^{-1}$ was determined after 3 days of incubation. Data are means with standard error in parentheses from at least two independent cultures assayed in triplicate. Cells were grown microaerobically in BVMN medium with different Cu concentrations.

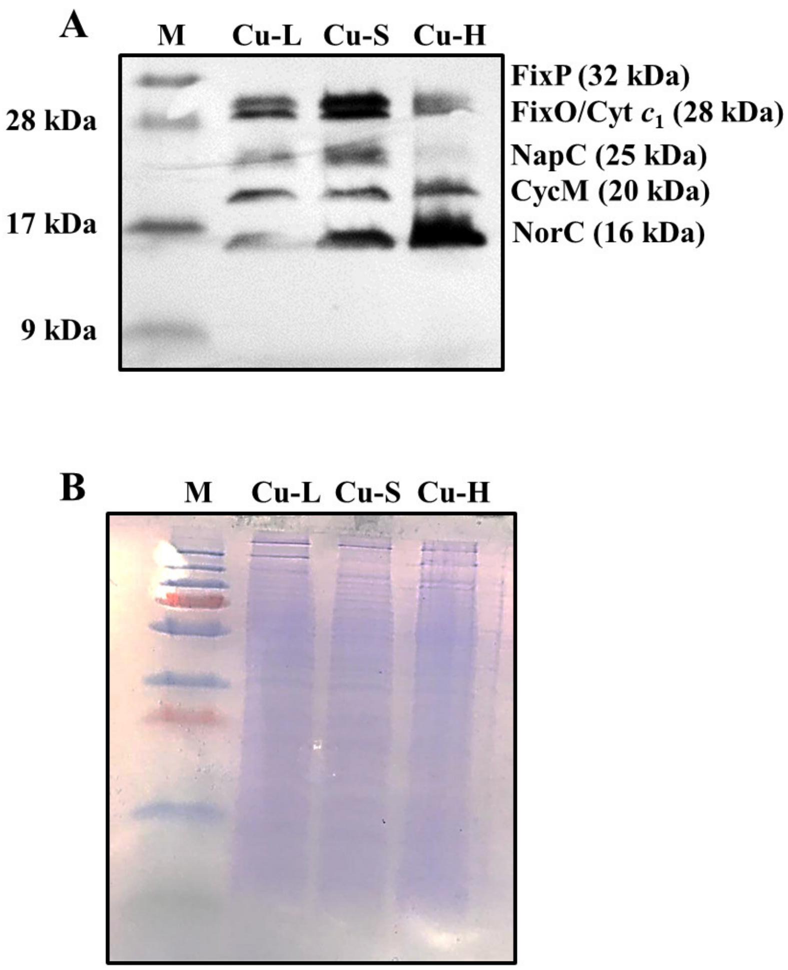

Figure S6. (A) Full scan of the entire gel of the haem-staining experiment shown in Figure 5A. Heme-stained *c*-type cytochromes are specified at the right margin. Apparent masses of the marker proteins (kDa) are shown at the left margin. **(B)** Coomasie® blue-stained SDS-PAGE gel made to visualize protein levels loaded in each lane for these experiments. M, protein marker ladder.

Table S1. Primers used in the qRT-PCR experiments.

Primers	DNA Sequence (5'→3')
bsr7036 (*napE*)_for_4	GCCTTCCTGTTCCTGAC
bsr7036 (*napE*)_rev_4	CCGGCAAACATCTGGTAGA
*nirK*_6_for	AGCCTTCACCGACACCGAAGAG
*nirK*_6_rev	GAGCGCATTCTTGCCGGTAAGC
*norC*_3_for	GCAGATGCCGCAGTTCAAC
*norC*_3_rev	TGATCGTGCTCACCCATTG
*nosR*_qRT-PCR_F	ATGATCCAGGTGCGGCTGAAG
*nosR*_qRT-PCR_R	CCGGCTGTGATGATTGTGTTCG
16S_qRT_For	GCAGGCTTAACACATGCAAGTC
16S_qRT_Rev	AGGTACGTTCCCACGCGTTACTC
